# Knowledge and awareness of and perception towards cardiovascular disease risk in sub-Saharan Africa: A systematic review

**DOI:** 10.1371/journal.pone.0189264

**Published:** 2017-12-12

**Authors:** Daniel Boateng, Frederick Wekesah, Joyce L. Browne, Charles Agyemang, Peter Agyei-Baffour, Ama de-Graft Aikins, Henriette A. Smit, Diederick E. Grobbee, Kerstin Klipstein-Grobusch

**Affiliations:** 1 Julius Global Health, Julius Center for Health Sciences and Primary Care, University Medical Center Utrecht, Utrecht University, Utrecht, The Netherlands; 2 School of Public Health, Kwame Nkrumah University of Science and Technology, Kumasi, Ghana; 3 African Population and Health Research Center, Nairobi, Kenya; 4 Department of Public Health, Academic Medical Center, University of Amsterdam, Amsterdam Public Health Institute, Amsterdam, The Netherlands; 5 Regional Institute for Population Studies, University of Ghana, Legon, Ghana; 6 Division of Epidemiology & Biostatistics, School of Public Health, Faculty of Health Sciences, University of the Witwatersrand, Johannesburg, South Africa; The Chinese University of Hong Kong, HONG KONG

## Abstract

**Introduction:**

Cardiovascular diseases (CVDs) are the most common cause of non-communicable disease mortality in sub-Saharan African (SSA) countries. Gaps in knowledge of CVD conditions and their risk factors are important barriers in effective prevention and treatment. Yet, evidence on the awareness and knowledge level of CVD and associated risk factors among populations of SSA is scarce. This review aimed to synthesize available evidence of the level of knowledge of and perceptions towards CVDs and risk factors in the SSA region.

**Methods:**

Five databases were searched for publications up to December 2016. Narrative synthesis was conducted for knowledge level of CVDs, knowledge of risk factors and clinical signs, factors influencing knowledge of CVDs and source of health information on CVDs. The review was registered with Prospero (CRD42016049165).

**Results:**

Of 2212 titles and abstracts screened, 45 full-text papers were retrieved and reviewed and 20 were included: eighteen quantitative and two qualitative studies. Levels of knowledge and awareness for CVD and risk factors were generally low, coupled with poor perception. Most studies reported less than half of their study participants having good knowledge of CVDs and/or risk factors. Proportion of participants who were unable to identify a single risk factor and clinical symptom for CVDs ranged from 1.8% in a study among hospital staff in Nigeria to a high of 73% in a population-based survey in Uganda and 7% among University staff in Nigeria to 75.1% in a general population in Uganda respectively. High educational attainment and place of residence had a significant influence on the levels of knowledge for CVDs among SSA populations.

**Conclusion:**

Low knowledge of CVDs, risk factors and clinical symptoms is strongly associated with the low levels of educational attainment and rural residency in the region. These findings provide useful information for implementers of interventions targeted at the prevention and control of CVDs, and encourages them to incorporate health promotion and awareness campaigns in order to enhance knowledge and awareness of CVDs in the region.

## Introduction

Non-communicable diseases (NCDs) pose a major health challenge globally, currently causing more deaths than all other causes combined.[1] In 2012, about 38 million people died from NCDs and this is expected to increase to 52 million by 2030.[1] About 80% of these deaths are caused by four NCDs: cardiovascular diseases (CVDs), cancers, chronic respiratory diseases and diabetes. CVDs account for almost half of NCDs deaths,[1,2] estimated at an annual 17.3 million deaths, and 10% of the global disease DALY burden.[2,3] It is expected that by the year 2030, more than 23 million deaths will be caused by CVDs,[3,4] with stroke and coronary heart disease (CHD) being the leading contributors.[5,6]

Deaths from CVDs have declined progressively over the past three decades in high-income countries because of implementation of population-wide preventive strategies, effective primary and secondary preventive healthcare, and availability of improved treatment for acute events.[7] However, rates of CVD deaths have increased in LMICs over the same period.[8,9] In addition to increased prevalence of risk factors of CVDs in these settings, this rise in CVD deaths reflects lower availability of population strategies for prevention and health care.[1] The rise in CVD risk factors in sub-Saharan Africa (SSA) is attributed to rapid urbanization, globalization and urban poverty.[10] Both are associated with a change in diets and lifestyle, where traditional diets are replaced with energy-dense and processed foods and increasing physical inactivity.[10] As poverty and inequality trigger the upsurge of communicable diseases,[11] as well as propagate risk factors for NCDs as smoking, drinking and poor diet,[11] the burden of disease disproportionally affects the urban poor.

Gaps in knowledge of CVD conditions and their risk factors in the general population are important barriers in the effective prevention and treatment of CVDs.[12] The role of knowledge in health behaviours and sustained behavioural changes has been proposed by several models including the health belief model.[13–15] This models posit that knowledge of a disease condition influences patient’s attitude and practice, improves compliance with treatment and has been shown to lead to reduction in prevalence and aversion of complications.[16] These models, although they may differ in content and viewpoint, emphasize the importance of appraising the beliefs, views and attitudes of individuals to apprehend observed behaviours and to guide behavioural change.

Success in the implementation of any health promotion program is dependent on context-specific information on knowledge, awareness and perception of the targeted population. There is however a regional level scarcity of evidence on the knowledge and awareness levels of CVDs and risk factors among the populations of SSA.[17] This systematic review therefore aims at synthesizing existing evidence on knowledge, awareness and perception towards these conditions.

## Methods

This review was conducted according to the recommendations outlined in the PRISMA (Preferred Reporting Items for Systematic Reviews and Meta-Analyses) statement.[18] (S1 File). It was registered with Prospero (CRD42016049165).

### Search strategy

We searched PubMed, Medline, Science Direct, Google Scholar, Africa Index Medicus (AIM), Africa Journals Online (AJOL) databases to retrieve relevant primary studies conducted in SSA, using pre-defined search (Title/Abstract) and indexing terms (MeSH/Emtree). Keywords and MeSH terms and their combinations used in the searches were “knowledge”, “stroke”, “heart attack”, “coronary heart disease”, “myocardial infarction”, “congenital heart disease”, “heart diseases”, “vascular diseases”. Reference lists of full-text papers were hand searched for additional articles and reviewed for relevance in this review. The strategy is provided as a supplementary file (S1 Text).

### Inclusion criteria

We included studies that were published in SSA, in English, and in peer-reviewed journals between 2007 and 2015. Papers were from primary research of any design and methodology: quantitative and qualitative and exploring knowledge, awareness and perception of CVD and the risk factors. Studies that were carried out among SSA populations living in Western countries or only described interventions leading to increased knowledge and awareness of CVDs or risk factors and symptoms of CVDs were also excluded.

### Definition of terms/concepts

CVDs include vascular diseases in general, CHD, cerebrovascular disease (e.g. stroke), myocardial infarction (MI) and congenital heart diseases. Individuals were required to correctly identify CVD conditions, risk factors and clinical symptoms from a list to gauge their knowledge. Perception was based on individuals’ self-assessment of chances of developing CVDs, as well as their understanding of who was at risk to develop the condition. Perception was mostly explored in qualitative studies. The SSA region was classified based on the United Nations classification of countries.[19]

### Data extraction

Two reviewers (DB, FW) conducted data extraction from the identified studies. Information was extracted on: authors, year of publication, study design and population, research methods, types of CVDs studied, findings on the knowledge, awareness of and perception towards CVDs and the risk factors. We extracted additional data on the factors influencing knowledge and perceptions of CVD and the reported sources of information on CVD and risk factors. The exercise was reviewed by JB and KKG, who were also consulted on the extraction process.

### Quality assessment

The quality of the quantitative studies, were assessed based on National Institute of Health (NIH) Quality Assessment Tool for Observational Cohort and Cross-Sectional Studies.[20] This form appraised the reliability, validity and generalizability of the quantitative studies. The NIH quality assessment tool uses 13 criteria to assess and rate the quality of studies. This included the research question, study population, sample size estimation, exposure and outcome assessment, loss to follow-up and statistical analysis. General guidance is provided for determining the overall quality of the studies and to grade their level of quality as good, fair or poor.

Qualitative studies were appraised using the Critical Appraisal Skill Programme (CASP) tool.[21] The CASP tool has 10 items that look at the relevance and clarity of research goals, appropriateness of the research design and methodology in addressing the research question, recruitment strategies, data collection, data analysis, findings, ethical consideration and value of the research. Questions attached to these items enable critical self-reflection about biases and assess the extent to which findings from the study could be transferred to other settings or groups. The quality assessment and criteria are available as a supplementary file (S2 File).

### Synthesis of findings

Qualitative data synthesis of the findings on the knowledge, awareness of and perception towards CVD risk and risk factors in SSA was conducted. Findings from the quantitative papers were absorbed using the multi-source synthesis method, an analytical technique that enhances transparency when synthesizing quantitative and/or contextual data, thus providing a platform for comparison between studies.[22] Findings from qualitative articles were integrated with those from the quantitative studies based on similar themes or topics. Due to the heterogeneity in outcomes, data were not pooled to conduct a meta-analysis.

## Results

### Study characteristics

**Fig 1:**
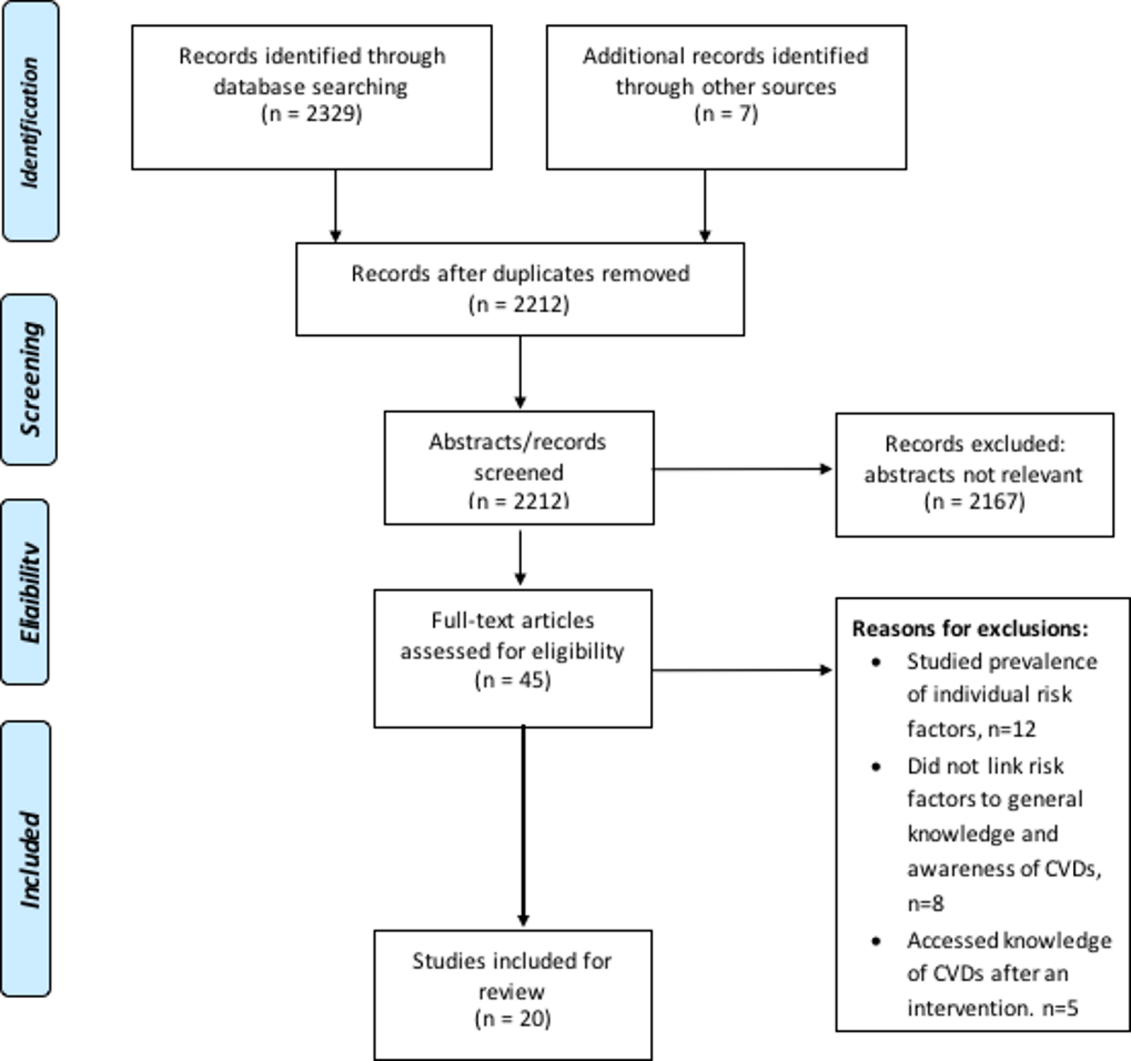
Flow chart of inclusion and exclusion of relevant articles.

A total of 2212 titles were identified from electronic database searches. 2167 titles were excluded for being irrelevant to the review question, and 45 full-text articles were assessed for inclusion. Twenty-five articles were excluded based on reasons such as not reporting the link between risk factors to general knowledge and awareness of CVDs or reporting results of an impact of an intervention in the levels of knowledge and awareness of CVD and risk factors. In the end, 20 articles were included in the review. The assessment and inclusion criteria are reported in Fig 1. One of the 18 quantitative studies out of the final 20 studies was quasi experimental, while the rest were cross-sectional. Respondents were recruited from varied settings, including from general population samples living in urban and rural areas, and from specific samples like academic staff, hospital staff and health professionals, patients, and employees in banks and in the military. The age of the participants in the different studies ranged from 16 to 82 years. More information on characteristics of study participants is presented in Table 1.

**Table 1:** Characteristics of included studies.

Study, year, country	Design and methods	Sample size	Study population and setting	Quality^†^
**Akintunde et al (2015)[23]; Nigeria**	Study design: Descriptive cross-sectional. / Methods: Quantitative; random sampling	206 (M 96, W110)	Adult university staff (academic and non-academic); Mean age 45.3years	Fair
**Mohammed (2012)[26] Nigeria**	Study design: Cross-sectional. / Methods: Quantitative. / Sampling: Not stated	82 (M 80; W 2)	Military personnel (Army, Navy, Air force) of the Nigerian Armed Forces; 30-60years (mean 49years)	Poor
**Uchenna, Ambakederomo, Jesuorobo (2012)[42]; Nigeria**	Study design: Cross-sectional. / Methods: Quantitative; convenient sampling	236 (M 136, W 100)	Outpatients of university teaching hospital; 16–82 years (mean 42.1years)	Poor
**Awosan et al (2013)[25] Nigeria**	Study design: Cross-sectional. / Methods: Quantitative, multistage random sampling	210 (M 141; W 69)	Bankers and secondary school teachers (>1yr experience) in a metropolis; 25-56years teachers; 20-49years bankers	Good
**Oladapo et al (2013)[33] Nigeria**	Study design: Cross-sectional survey. / Methods: Quantitative. / Sampling: Systematic random	2000 (M 873; W 1127)	Rural community members in Southwestern Nigeria	Good
**Akinyemi et al (2009)[28],** **Nigeria**	Study design: Cross-sectional survey. / Methods: Quantitative, systematic random	400 (M 137; W 233)	Hospital staff of federal medical centre; 20-64years (mean age 34.4years)	Fair
**Wahab, Kayode & Musa (2015)[37] Nigeria**	Study design: Cross-sectional survey. / Methods: Quantitative	354 (M 148; W 166)	Patients on follow-up for hypertension and/or diabetes at specialist medical outpatient clinics; Mean age 56.4years	Good
**Obembe et al (2014)[29] Nigeria**	Study design: Cross-sectional survey. / Methods: Quantitative, multistage stratified sampling	494 (M 284; W 210)	Staff of government-owned tertiary institution	Good
**Komolafe et al (2015)[31] Nigeria**	Study design: Cross-sectional survey. / Methods: Quantitative	Size: 114 (M 51; W 63)	Secondary school teachers of 2 towns in Nigeria; 20-50years	Fair
**Ajayi and Ojo (2007)[40]**	Study design: Descriptive cross-sectional. / Methods: Quantitative	155 (M 87; W 68)	Patients attending a medical out-patient clinic; Mean age 58.4	Poor
**Akinyemi RO et al (2015)[30]^§^ Nigeria**	Study design: Quasi experimental. / Methods: Quantitative	116 (M 50; W 66)	Non-neurologist health workers; Mean age 46.1	Fair
**Ansa, Oyo-Ita and Essien (2007)[17] Nigeria**	Study design: Cross-sectional. / Methods: Quantitative; systematic random sampling	500 (M 302; W 198)	Staff of university hospital; 41-50years	Fair
**Donkor et al (2014)[35] Ghana**	Study design: Cross-sectional survey. / Methods: Quantitative, systematic random	693 (M 374; W 319)	Inhabitants of a metropolitan city, Mean age, 36.8years	Good
**Cossi et al (2012)[36] Benin**	Study design: Cross-sectional survey. / Methods: Quantitative. / Sampling: All included	15155 (M 6293; W 8862)	Adults in an urban district; Mean age, 31years	Good
**Kaddumukasa et al (2015)[32]** **Uganda**	Study design: Cross-sectional survey. / Methods: Quantitative multistage stratified random	370 (M 117; W 253)	Households in selected urban and rural areas; 18-85years; Median age, 34years	Good
**Nakibuuka et al (2014)[24] Uganda**	Study design: Cross-sectional. / Methods: Quantitative; multistage stratified sampling. / Analysis: Chi square, logistic regression	1616 (M 510; W 1,106)	Urban and rural residents; 1161 urban, 455 rural; Mean age 39.6	Good
**Temu et al (2015)[41] Kenya**	Study design: Cross-sectional. / Methods: Quantitative; convenient sampling	300 (M108; W 192)	PLWH on or not yet on ART (outpatients) from HIV clinic of Teaching and referral hospital; > = 18years	Good
**Yuqiu & Wright (2008)[34] South Africa**	Study design: Cross-sectional survey. / Methods: Quantitative, census sampling	551 (M 302; W 249)	Adults of working age living in a community; 18-40years	Fair
**Qualitative study**				
**Surka et al (2015)[27] South Africa**	Study design: Cross-sectional. / Methods: Qualitative (FGDs of 8–10 participants); Purposive sampling	28 (M 4; W 24)	Male and female community members (≥ _25 years) with no previous experience in being assessed for CVD risk; Mean age 53years	Good
**Awah et al (2008)[39] Cameroon**	Study design: Cross-sectional. / Methods: Qualitative (FGDs and IDI); Purposive sampling;	82 (M 44; W 38)	Community members, health workers, policy makers	Good

* HDFQ = Heart Disease Fact Questionnaire; CVD = cardiovascular disease; IHD = ischemic heart disease; PLWH = People living with HIV/AIDS; CHD = Coronary Heart Disease; BP = Blood pressure; PLWH = People living with HIV/AIDS;

^**†**^The quality assessment and criteria are available in the S2;

^§^Only those in the pre-intervention phase included in this review; FGD = Focus group discussions; IDI = In depth interviews

### Quality of included studies

The majority of the quantitative studies were rated to be of good or high quality (n = 10). They described in detail the design and methodology used, the process of recruiting participants, justification and methods of arriving at required sample size, study setting, clear and detailed presentation of findings. Studies that were rated to be of fair or poor quality (n = 8) were papers that failed to describe details of subject recruitment processes including inclusion criteria and sampling strategies and lacked justification of sample size and other issues that could lead to a high risk of bias and undermine generalizability of the study (S1 File).

### Knowledge and awareness regarding cardiovascular diseases

Most studies in this review did not state a priori the criteria used in measuring and classifying levels of knowledge and awareness. However, most of them classified knowledge and awareness of CVD or the risk factors as poor, acceptable or good. In the study by Akintunde et al,[23] among university staff, a knowledge score of <50% was classified as low; 50–69% moderate and ≥70% good. Nakibuuka et al[24], in a study in Uganda classified urban and rural residents who could identify 5–10, 2–4 and <2 CVD risk factors or warning signs as having good, fair and poor knowledge respectively.

**Fig 2:**
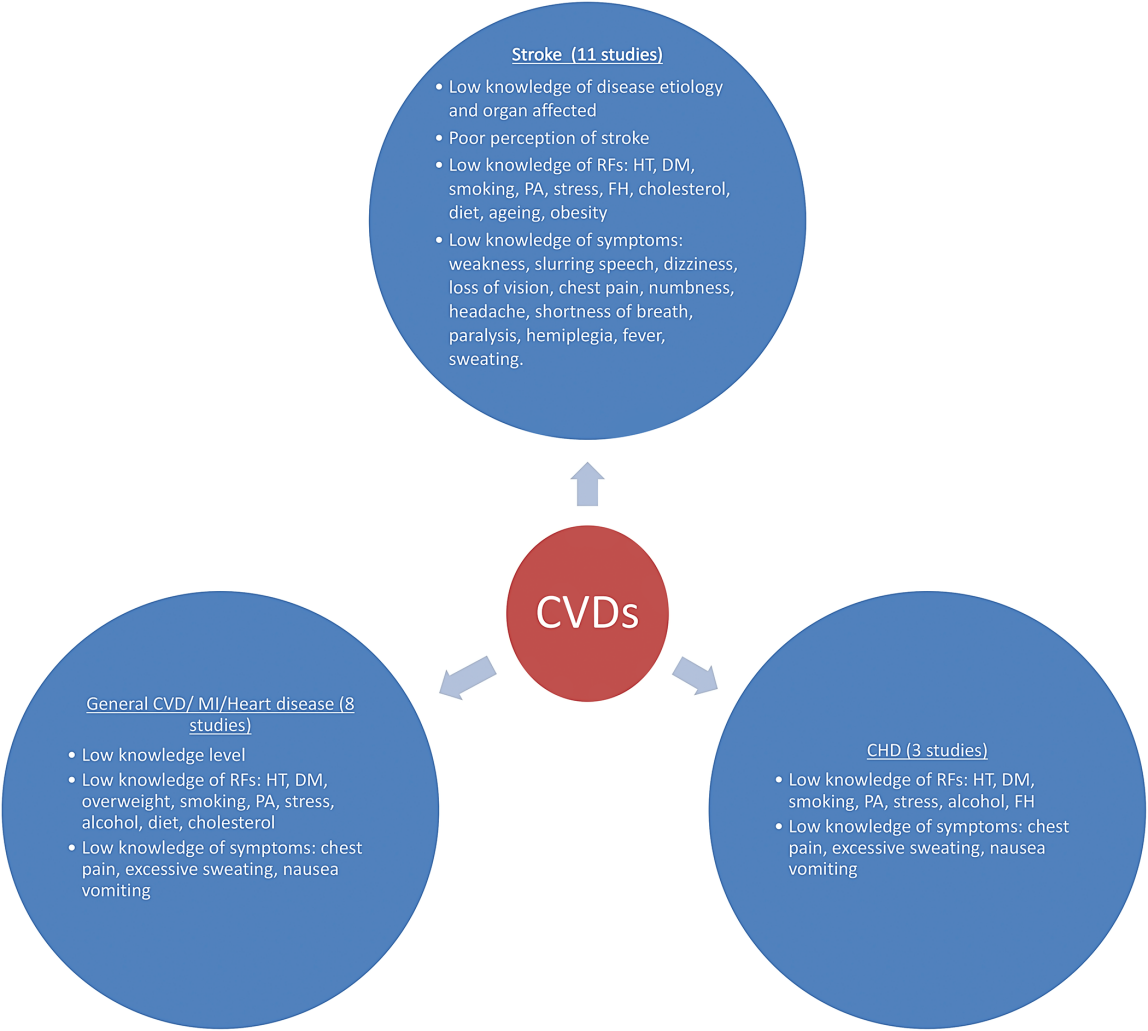
Summary of results. RF, Risk factors; HT, Hypertension; DM, Diabetes Mellitus; PA, Physical activity; FH, Family History; CVD, Cardiovascular disease; MI, Myocardial infarction.

Awareness of CVDs was high among studies that reported on it; 76.2% among bankers and teachers[25] and 75.6% among military personnel[26] in Nigeria. Most people in a low-income peri-urban community in South Africa,[27] were familiar with the terminology used to describe CVDs. However, the studies reported generally low knowledge levels of CVDs with most studies reporting less than 50.0% of respondents having good knowledge. In studies conducted among workers in a Nigerian University Hospital, one reported that 19.0% had good knowledge of CVDs[23] while another showed that 53.5% knew the mechanism through which stroke occurs.[28] Findings on the knowledge and awareness of CVDs in SSA is summarized in Table 2 and Fig 2.

**Table 2:** Outcome assessment and findings of included studies.

Study	CVDs studied	Assessment of knowledge	General knowledge/ awareness of CVDs	Knowledge of risk factors	Knowledge of warning signs/symptoms	Factors related
**Akintunde et al [23]**	CVD	HDFQ scores were used to determine the level of knowledge	50% had low knowledge of CVDs; 31.1% moderate; 19.9% high.	Poor knowledge on cholesterol and heart disease. / Moderate knowledge of smoking, diabetes, overweight and high BP.		Age, gender and education **not** associated with knowledge of CVDs.
**Uchenna, Ambakederomo, Jesuorobo[42]**	CVD	Structured questionnaire- researcher administered	91.2% never been counselled on heart disease prevention.	51.7% had no knowledge of any cause of heart disease.	Low knowledge of symptoms of heart disease; 24.6%	Education; gender **not** associated with awareness of CVDs.
**Mohammed [26]**	CVD	Self-designed knowledge and awareness Questionnaire	75.6% enlightened on CVD.	Low knowledge level of; ➢Primary risk factors; 31.7% ➢Secondary risk factors; 41.5% ***Identified risk factors***: Smoking, 70.6%; excessive alcohol, 52.8%; stress, 87.5%; sedentary lifestyle, 16.6%; poor dietary intake, 6.4%.		
**Awosan et al[25]**	CHD	Questionnaire adapted from the American Heart Association’s questionnaire	High level of awareness of CHD, 76.2%.	Up to 50% knew 4/7 risk factors among teachers and 1/7 among bankers. / ***Identified risk factors;*** Hypertension, 50.5% teachers and 59% bankers; overweight/obesity, 47.6% teachers and 55.2% bankers; physical activity, cigarette smoking and fatty foods; up to 50% among teachers; less among bankers.		
**Oladapo et al [33]**	Stroke Heart failure	Structured questionnaire	Low knowledge of clinical features of stroke 21.9% and heart attack or angina, 0.4%.	56% unable to identify a single risk factor ***Identified risk factors***: Hypertension, 16.2%; diabetes, 5.4%; tobacco use, 36.2%; obesity, 1.6%; lack of exercise, 1.2%; stress (42.7%).		Age, gender, family history, history of stroke **not** related Tertiary education (OR, 95% CI = 3.11, 2.06–7.14).
**Akinyemi et al [28]**	Stroke	Structured, semi-closed questionnaire	Knowledge of organ affected by stroke; 16.9% among clinical workers; 35.0 among non-clinical workers. / Knowledge of mechanism through which stroke occurs; 53.5%. / Knowledge of occurrence of stroke through rupture of vessels; 64.9%.	4.3% could not identify a single risk factor; 27.6% identified 1–3 risk factors; 68.1% identified > = 4 risk factors ***Identified risk factors*:** Hypertension, 88.6%; stress, 70.8%; high cholesterol, 43.8%; alcohol consumption, 43.4%, Smoking, 35.9%, lack of exercise, 34.6%, ageing 30.8%; diabetes 29.5%; bad diet 22.4%. */ Some participants cited evil spirits*, *13*.*8% and will of God*, *1*.*1%*.	8.6% could not identify a single warning symptom. / ***Identified symptoms***: One sided body weakness (most identified), 61.9%; slurring of speech, 52.2%; dizziness, altered consciousness, loss of vision, chest pain least identified.	Tertiary education demonstrated better knowledge of how stroke occurs (p<0.001). / Tertiary education knew < = 4 warning symptoms (p<0.004).
**Wahab, Kayode & Musa [37]**	Stroke	Author designed questionnaires		Only 39.8% correctly mention 1 modifiable stroke risk factor. / ***Identified risk factors*:** Hypertension, 34.7%; diabetes, 7.3%; smoking, 3.8%; alcohol, 4.5%; stress, 12.7%; overweight/obesity, 1.9%; Sedentary lifestyle, 0.6%.		Age < 55 years (OR, 1.832; 95% CI, 1.160–2.893); >12 years of formal education (OR, 2.712; 95% CI, 1.678–4.382); family history (OR, 2.112; 95% CI, 1.116–3.998); urban residence (OR, 2.726; 95% CI, 1.256–5.919).
**Obembe et al [29]**	Stroke	Author designed questionnaires		1.8%% knew **no** risk factor ***Identified risk factors***: Hypertension (most identified), 91.7%; stress, 80.2%; ageing, 63.8%; cholesterol, 51.4%; smoking, 46.2%; obesity, 56.1%; lack of exercise, 50.8%; family history, 55.5%; diabetes 45.7%; alcohol, 40.3%; diet, 36.0%.	7.7% identified **no** warning sign; only 15.2% identified all warning signs. / ***Identified symptoms***: Slurred speech, 58.7%; dizziness, 52.8%; numbness, 69.4%; weakness, 69.8%; headache, 39.9%; vision problem, 39.5%; difficulty in understanding, 34.4%.	Age, education, family history significantly influenced awareness of stroke.
**Komolafe et al [31]**	Stroke	Previously validated questionnaire to recognize and identify risk factors and early warning signs	Inadequate awareness of stroke.	13.2% identified **no** risk factor. ***Identified risk factors***: Hypertension, 79.8%; ge, 43.9%; Stress, 65.8%; cholesterol, 50.9%; obesity, 49.1%; lack of exercise, 57%; family history, 52.6%; diabetes, 47.4%; alcohol, 52.6%; diet, 99.1%; hyperlipidemia, 22.8%; smoking, 49.6%; ageing, 43.9%.	23.7% identified **no** warning sign; only 3.5% identified all warning signs. ***Identified warning signs*:** Slurred speech, 50%; dizziness, 27.2%; numbness, 33.3%; weakness, 42.1%; headache, 36.8%; vision problem, 20.2%; shortness of breath, 32.5%.	
**Donkor et al [35]**	Stroke	Author designed, validated questionnaire, based on previously used questionnaires	Inadequate awareness of stroke.	19% identified **no** risk factor. ***Identified risk factors***: Lack of exercise, 37%; hypertension, 34%; alcohol 33%; high cholesterol, 32.0%; family history 28%; smoking, 24%; stress 22%; (heart disease, obesity, diabetes) <15%.	22% identified **no** warning sign. ***Identified warning signs*:** Slurred speech, 37%; dizziness, 17%; numbness, 21%; weakness, 38%; severe headache, 25%; vision problem, 15%; shortness of breath 13%.	Age, gender, education not related to stroke awareness.
**Ajayi and Ojo (2007)[40]**	Stroke	Structured questionnaire- researcher administered		***Identified risk factors***: Hypertension (most identified), 60.6%; previous history, 16.1%; cholesterol, 3.2%; family history, 3.2%; smoking, 1.3%. / *None identified drinking of alcohol as risk factor*.	***Identified warning signs*:** Paralysis on one side of body, 55.6%; weakness on one side, 27.1%; sudden difficult in speaking and understanding, 7.1%; tingling sensation, 5.8%; blurred vision, vertigo, difficulty swallowing <1%. / None identified chest pain.	Higher education association with increase awareness of stroke risk factors.
**Akinyemi RO et al[30]§**	Stroke	Self-administered questionnaire.	Knowledge of epidemiology of stoke, 81%.	90.5% identified > = 4 risk factors; 7.8% identified 1–3 risk factors; 1.7% identified no risk factor. / 95.7% identified hypertension as major risk factor.	79.3% identified > = 4 risk symptoms; 19% identified 1–3 symptoms. / ***Identified warning signs*:** Face drop, 11.2%; arm weakness, 12.1%; slurred speech 18.1%.	
**Ansa, Oyo-Ita and Essien[17]**	IHD	Self-administered questionnaire		***Identified risk factors***: Smoking, 70.6%; excessive alcohol, 52.8%; obesity, 41.6%; sedentary lifestyle, 16.6%; oral contraceptives, 6.4%.		Higher education increased knowledge of risk factors.
**Cossi et al [36]**	Stroke	Author designed semi-structured questionnaires adopted from previous studies	Majority were unable to name organ affected by stroke.	21.8%% knew **no** risk factor. ***Identified risk factors***: Hypertension most identified, 34.5%; Stress, 7.6%; diet, 4.7%; diabetes, 0.3%; cardiac problems, 0.3%; obesity, 1%.	22.7% knew no warning sign of stroke; 33% knew > = 1 warning sign. ***Identified warning signs*:** Paralysis and hemiplegia, 34.4%, Weakness, walking in speaking and seeing 12.8%; Headache and dizziness, 11.8%.	Education, age, occupation associated with knowledge of stroke risk factors.
**Kaddumukasa et al[32]**	Stroke	Modified standardized questionnaire already used in SSA settings	59.4% did not know brain as site affected by stroke.	42.4% knew **no** risk factor. ***Identified risk factors***: Stress (most identified), 43%; hypertension, 28.9%; (Age, diabetes, fats, diet, lack of exercise), <10%. / None identified smoking	57% knew **no** warning sign. ***Identified warning signs*:** Paralysis (most identified), 18%; Body weakness, 12%; numbness 10%.	Residence associated with knowledge of stroke (p = 0.038)
**Nakibuuka et al[24]**	Stroke	Structured questionnaires, modified from previous studies	76.2% urban, 78.9% rural did **not** know organ affected by stroke. / Some believe stroke affect the heart, liver and kidneys. / 39.5% knew stroke is preventable.	73% knew **no** stroke risk factor. ***Identified risk factors*:** hypertension, 56%; stress, 51.4%; bad diet, 29.6%; lack of exercise 25.7%; diabetes, 14.9%; old age, 12.6%; High cholesterol, 12.2%; obesity, alcohol <10%; smoking, 0.7%. ***/*** *Some cited demons or witchcraft*, *0*.*9%; God’s will*, *6%*.	75.1% knew **no** stroke symptom; 40.3% only 1 symptom; Only 3% knew ≥ 5 symptoms. ***Identified symptoms***: paralysis on one side, 28.6%; weakness on one side, 26.1%; dizziness, 23.6%; paralysis on any part, 17.4%; tiredness, 16.4%; headache 16.2%; shortness of breath, fever/sweating, 9.7%; weakness on any part of body, 7.5%, blackout, 6.5%; blurred vision, 2.7%; speaking difficulty, 2.2%.	Tertiary education associated with good knowledge of; -risk factors, (OR 5.96; 95% CI 2.94–12.06). Warning symptoms (OR 4.29; 95% CI 2.13–8.62). Urban residence increased knowledge or CVD.
**Temu et al [41]**	CVD; CHD	Questionnaire constructed from multiple validated surveys. / Knowledge measured on a continuous scale and scored		Mean knowledge score 1.3/10. ***Identified risk factors*:** Stress, 74%; obesity, 9.3%; raised BP, 9%; excessive alcohol, 7.6%; smoking, 4%; age, 2.3; family history 1.3%.	Mean knowledge score 0.28/7. 77.3% didn’t know heart attack. / <3% could identify chest pain, excessive sweating, nausea vomiting, pain in teeth, jaw or arm as symptoms.	Education → (OR 5.21, 95% CI 0.99–27.37)
**Yuqiu & Wright [34]**	CVDs	Author designed questionnaire	Generally low knowledge of CVDs.	***Identified risk factors***: Stress, 53.5%; physical inactivity obesity, alcohol, <30% in males and females) and diabetes (<10%).		
**Surka et al [27]**	CVD, heart attack, stroke, MI	Thematic discussions on Knowledge of CVD and its prevention, perception of risk	Majority familiar with terminologies for CVDs; Limited insight into the conditions.	***Cited risk factors for CVD*:** Tobacco smoking, excessive alcohol consumption, stress, unhealthy diets.		
**Awah et al[39]**	CVD	FGD and IDI guides		***Perceived risk factors of CVDs***: Diet, obesity, smoking, alcohol, sedentary lifestyle.		

BP = Blood pressure; MI = Myocardial infarction

### Knowledge of risk factors for cardiovascular diseases

To gauge knowledge of risk factors for CVDs, individuals were required to correctly identify them from a list. Just like it was the case with CVD risk, majority of the studies also reported low levels of knowledge on risk factors for CVDs. Hypertension and stress were the most known and cited risk factors in most of the studies. Participants who were unable to identify a single risk factor for CVDs ranged from as low as 1.8% in a study among hospital staff in Nigeria[29] to a high of 73.0% in a population-based survey in Uganda.[24] Specifically, among studies that looked at stroke, participants who could not identify a single risk factor was <20% among hospital workers,[28,30]university staff[29] and secondary school teachers[31] in Nigeria and 40–80% among rural and urban Ugandans[24,32] and rural Nigerians.[33] A study that looked into coronary heart disease among teachers and bankers in Nigeria also described about 20% of the study population as having no knowledge of risk factors for the disease.[25] The studies also reported some misconceptions regarding the risk factors for CVDs to include evil spirits, demons and will of God as causes of CVDs.[24,28]

### Hypertension

Knowledge levels of hypertension as a risk factor for CVD ranged from as low as 16.2% in a study among rural community members in Nigeria[33] to 95.7% in a study among health workers in Nigeria.[28] In a low-income peri-urban community in South Africa, none of the respondents cited hypertension as a risk factor of CVD.[27] Low knowledge levels of hypertension as risk factor for CVDs, ranging from 16.2% to 34.5% were reported among studies conducted within urban and rural communities[32–36] whereas high percentages were reported in studies conducted among health workers (95.7%);[30] (88.6%),[28] secondary school teachers (79.8%)[31] and staff of tertiary institution (91.7%).[29]

### Diabetes

The knowledge level of diabetes as a risk factor of CVD ranged from 0.3% in a study among urban adult population in Benin[36] to 47.4% among secondary school teachers in Nigeria.[31] Two community-based studies from Ghana[35] and Uganda[24] reported less than 15% of study participants possessing any knowledge of diabetes as a risk factor for stroke. Knowledge of diabetes as a CVD risk factor among hypertension and diabetes patients at a specialist medical centre in Southern Nigeria was very low, at 7.3%.[37]

### Smoking

Knowledge of smoking as a CVD risk factor was 70.6% among military personnel in Nigeria[26] and less than one percent among the general populations in Central Uganda.[24] Less than 50% of respondents across all studies could identify smoking as a risk factor for CVD, with the exception of the study among Armed Forces personnel in Nigeria, 70.6%.[26] In a study in rural Uganda, none of the respondents identified smoking as a risk factor for CVD.[32] In all, 14 studies reported on knowledge of smoking as CVD risk factor, three of which reported <5% with knowledge of smoking as a risk factor for stroke^26,42^ and for CHD.[38]

### Physical inactivity

Knowledge of physical inactivity or sedentary lifestyle as risk factors for CVD ranged from 0.6%[37] to 57%,[31] in Nigeria. Two other studies reported knowledge level of less than 10%; 1.2% in a rural Nigerian community[33] and 3.8% among hospital outpatients.[37]

### Heavy alcohol consumption

Heavy alcohol consumption as a risk factor for CVD was reported by 4.5% in a study among patients with hypertension and/or diabetes at specialist medical outpatient clinics in Nigeria[37] to as high as 52.8% among staff at a University Hospital and same proportion among military personnel in Nigeria.[26] Another study among secondary school teachers[31] and Hospital staff in Nigeria[28] reported 52.6% and 43.4% of knowledge of alcohol as risk factor, respectively. Participants enrolled in qualitative studies conducted in South Africa and Cameroon[27,39] also mentioned heavy alcohol consumption as risk factor for CVD. Respondents in a study among outpatients in Nigeria were, however, not able to identify heavy alcohol use as a risk factor for CVD.[40]

### Stress

Stress was reported as a risk factor for CVD by 7.6% of adults in an urban district of Benin[36] to 87.5% of members of Nigerian Armed forces.[26] Other studies conducted among formally employed workers reported high knowledge level; health workers 70.8%,[28] among university staff 80.2%[29] and secondary school teachers 65.8%.[31] Among community level studies, knowledge level of 53.5% as among a South African community[34] whereas studies among urban communities in Ghana[35] and Benin[36] reported low knowledge of stress (22% and 7.6%) respectively.

### Other risk factors

Other risk factors for CVD were ageing, family history, obesity and unhealthy diet. Knowledge of these risk factors was low across studies reviewed and was least cited or known among study subjects. Ageing was identified as a risk factor for CVD by 63.8%, 43.9% and 38% among university staff,[29] secondary school teachers[31] and hospital staff[28] respectively in Nigeria. Among the studies that reported on family history, knowledge level was >50% in two of the studies that were conducted among formal working populations in Nigeria,[29,31] 3.2% among medical outpatients in Nigeria[40] and as low as 1.3% in the study conducted among people living with HIV/AIDS in Cameroon.[38] Of nine studies, five that were conducted among people living with HIV,[38] hypertension and/ or diabetes outpatients,[37] rural population,[33] urban population[36] and the general population,[24] <10% identified obesity as a CVD risk factor. The biggest proportion with knowledge of obesity as a CVD risk factor, 56.1% was reported among staff of a University in Nigeria.[29] Knowledge on diet as risk factor for CVD was 99.1% among secondary school teachers[31] and <10% among Armed Forces personnel[26] in Nigeria and the general household population in Uganda.[32] Unhealthy diet was also reported as a risk factor in two studies.[27,39]

### Knowledge of symptoms/ clinical signs of cardiovascular disease

The proportion of respondents who could not identify a single symptom of any CVD condition ranged from 7.0% among academic staff in a University in Nigeria[29] to 75.1% among the general population in Uganda.[24] The proportion of respondents who could identify all symptoms ranged from 3.5% among teachers[31] to 15.2% among health staff[29] in Nigeria. Knowledge of chest pain, excessive sweating, nausea, vomiting, and pain, as symptoms of CVDs were also very low (<3%).[38,40] Knowledge of symptoms of stroke was <50% in all the studies that reported on stroke with the exception of three, which reported >50% knowledge level of one (paralysis, 55.6%) among medical outpatients,[40] two (weakness, 52.2%; slurring speech, 61.9%) among hospital staff^31^ and four symptoms (slurring speech, 58.7%; dizziness, 52.8%; numbness, 69.4% and weakness, 69.8%) among university teachers.^32^ The most reported symptoms of stroke were weakness, 61.9%[28] and 69.8%,[29] slurring speech, 59%[29] and paralysis on one side, 55.6%.[40] Dizziness, loss of vision, chest pain and altered consciousness, headache, vision problem, shortness of breath and numbness were least reported across the studies.

### Perception of cardiovascular disease risk

Four studies investigated the perception of CVDs.[24,27,40,41] Among people living with HIV/AIDS, 31% believed they were at high risk of developing CVDs, while older women were more likely to agree that they were at a higher risk for CVDs.[41] In a qualitative study from South Africa,[27] participants were described as being generally unfamiliar with the concept of risk, while the two respondents who were familiar with the concept of risk could also not explain in detail what it actually meant. In a study of medical out-patients in a tertiary health institution in Nigeria,[40] majority (65.8%) of the respondents were never concerned about the possibility of developing stroke, 16.1% sometimes thought of it, 12.3% occasionally and 5.8% always had the concern. 34.1% of respondents in a population-based study from Uganda[24] perceived no chance while 14.4% perceived high chance of possible stroke in lifetime.

### Factors influencing knowledge of cardiovascular diseases and risk factors among reported studies

Factors such as age and family history, type of residence and education were reported to be associated with knowledge of CVDs. The significant influence of age on knowledge of CVD was reported by three studies.[29,36,37] In two studies from Nigeria conducted among hospital outpatients[37] and university staff,[29] age <55 and <40 were a significant predictor of knowledge of CVDs. There was a significant relationship between educational attainment and knowledge of CVDs.[17,29,33,36,37,40] As reported in a study from rural South-Western Nigeria,[33] people with tertiary education were three times more likely to be knowledgeable of CVD risk factors and a study among hospital outpatients in Nigeria[37] showed that more than 12 years of education increased the odds of being knowledgeable about CVD risk factors by more than twice. A significant association between type of residence and knowledge of CVD was also described: urban residents were more knowledgeable about CVDs compared to their rural counterparts in a community study in Uganda[24] and a study among diabetic/hypertensive outpatients.[37] No study reported a relationship between gender with knowledge of CVDs.[33,42]

### Sources of information on cardiovascular diseases

The sources of information for CVD and risk factors included electronic media like television,[25,26,31,36,41] radio,[26,31,41] and print media in the form of magazines or newspapers,[25,31,41] health care professionals[25,26,31,33,36,40,41] and family members or relatives.[25,31,33,36] The internet was reported as a source of information among secondary school teachers[31] and among people living with HIV/AIDS.[41] Television was the most cited source of CVD information across the studies that reported on it, with a proportion of 31.7% in a study of Nigerian Armed forces[26] to 75.5% in a study among University staff.[29] Healthcare professionals as source of CVD information ranged from 4%[41] to 64.4%[17] in people living with HIV and university staff respectively. In the study among hospital workers in Nigeria,[28] 66.9% and 23.2% of clinical and non-clinical staff had read on CVDs from other sources. Details of the sources of information reported across the studies are presented in Table 3.

**Table 3:** Sources of information about CVDs.

Source of information	Temu et al[41]	Mohammed[26]	Awosan et al[25]		Oladapo et al[33]	Akinyemi et al[28]		Komolafe et al[31]	Cossi et al[36]	Ansa, Oyo-Ita and Essien[17]
			Teachers	Bankers		Clinical	Non-clinical			
**Television**	51	31.7	53.8	43.8				75.4	13.9^*§*^	
**Radio**	44	12.2						56.1		
**Magazine/newspaper**	19		21.3	27.5				59.4		
**Internet**	4							40.4		
**Healthcare professional**	4	22.9	7.5	13.8	9.1			45.0	11.8	64.4
Media*					24.6	37.3	27.7			28.8
**Family**					59.9	30.3	20.5	27.3	25.1	
**Friend**						44.4	33.3			
**School education**						68.3	10.3	38.7	9.5	
**Seen someone with the condition**						81.0	82.0	16.6		
**Health campaigns**								33.8		
**Read from other sources**						66.9	23.2%		20.4	36.2

*Radio, public enlightenment programmes, and newspapers;

^§^Include radio and Internet

## Discussion

This review identified low levels of knowledge and awareness of CVDs and associated risk factors and clinical signs or symptoms for CVDs among populations in SSA. The knowledge gap is also apparent in the low perception regarding the risk of developing and dying from CVDs in the region.[24,40] In population-based studies conducted in Uganda[24] and Benin,[36] respondents were unable to identify the organ affected by stroke, despite it being a condition with poor survival outcomes in this region.[43–45] Knowledge of clinical symptoms was as low as 3.5% among teachers in Nigeria,[31] while as few as 16.2% in a rural Nigerian community[33] knew that of hypertension, 0.3% for diabetes and 1% for obesity and 7.6% for stress in Urban Beninese population,[36] as risk factors or developing CVD.

A systematic review of awareness of hypertension in West Africa reported overall low knowledge of hypertension.[46] Studies that explored knowledge and perceptions of obesity and sedentary lifestyles showed poor perceptions and subjective norms such as overweight being socially desirable, and a sign of beauty and riches thereby inducing unwillingness to lose weight.[47,48] African belief systems are however not static–they are complex and dynamic, tied as they are to shifting social identities. Other body of evidence suggests that contrary to the often-cited fatness equals wealth, health and beauty theory, young African women view fatness as a precursor for CVDs.[49] These women are interested in living a healthy life and are willing to reduce their body size in order to reduce the risk of obesity-related diseases despite the resistance to lose weight because of the cultural value on weight and the impact of the husband's preference.[50] These inherent perceptions and desire to lose weight should be important considerations when designing educational interventions to improve knowledge of CVDs.

Despite the rise in CVD risk factors in SSA populations, our findings indicate that the populations generally did not recognize their potential relation to the development of CVDs. In SSA, the incidence and prevalence of classical risk factors of CVDs such as smoking,[51] hypertension,[52] obesity,[53–55] high cholesterol, fatty diets, alcohol consumption[56–58] and lowered physical activity[59] are rising. This rise is linked to rapid urbanization, resulting in an epidemiological and nutrition transition, where energy-dense diets replace traditional diets and sedentary lifestyles prevail poverty.[10] As such, there is a shift in disease burden from under-nutrition and highly active lifestyle to over-nutrition-related and sedentary lifestyle related chronic diseases.

Knowledge of alcohol intake as a risk factor for CVD was low in the region. Four studies[24,34,37,41] reported on this and found that <30% of study participants cited alcohol consumption as a risk factor for CVDs; in a study among medical outpatients,[40] none identified alcohol consumption as a risk factor for CVD. In most societies in SSA, use of alcohol has been defined by cultural and religious parameters, with little acceptance of the potential health effect of alcohol consumption on health.[60] This is of concern, considering the expansion of alcoholic industries commercial activities in SSA to increase sales in this region.[61,62] Adequate policies to address these challenges in SSA are however few whereas there are no developed multi-sectorial approaches, that involves the private sector, civil society, informal sector, community leaders and traditional healers.[63] Further, in countries where there are preventive interventions such as enactment of drinking and driving laws, taxation, restrictions on advertising and community information, implementation is *ad hoc*, informal, fragmented and often lacks adequate control and enforcement systems.[63]

The relationship between alcohol consumption and CVDs is nuanced. Light to moderate drinking has been suggested to decrease the incidence of ischaemic stroke, whereas heavy drinking has been implicated as an independent risk factor for ischaemic and haemorrhagic stroke.[64–66] For hypertension, cardiac dysrhythmias and haemorrhagic stroke, alcohol is considered to be an independent risk factor, regardless of the drinking pattern.[67] This emphasizes the need for the development and enforcement of adequate and effective policy measures, public awareness and surveillance mechanisms in the SSA region. Without awareness of personal susceptibility and health consequences related to alcohol consumption, alcohol consumption behaviours are less likely to be modified to reduce risk of CVD.

Knowledge on stress as a risk factor of CVD was relatively high, especially among urban populations, despite the complex relationship between stress and CVDs.[68] Susceptibility to stress is influenced by type of personality, social support, coping strategies and genetic vulnerability.[68] Stress could be positive, by forcing us to adopt and thus to increase the strength of our adaptation mechanisms (eustress) or negative, when it exceeds our ability to cope, fatigues body systems and causes behavioural or physical problems (stressors).[68,69] A strong association has been observed between perceived stress and CHD[70–72] and current evidence shows perceived stress to be an independent risk factor for stroke.[73] The belief and perception of the influence of stress on CVDs in SSA populations could however be related to experiences of psychosocial stressors arising out of urbanization and poverty.[74,75] Experiences of chronic poverty-related stressors, such as inadequate housing, sanitation, water, overcrowding, environmental conditions, low education and unemployment, are potent predictors of poor cardiovascular health.[76–78] Strategies to deal with perceived psychosocial stress among these populations, include smoking and alcohol consumption, which themselves are precursors of poor cardiovascular health.[79,80]

This review shows knowledge of CVDs and their risk factors to be significantly related to the type of population studied and place of residence, and the level of exposure to health information about CVDs. Studies that formally tested the association between place of residence and education on knowledge of CVDs, also reported a significant relationship.[24,32,37] There is the possibility that the differences observed in the levels of knowledge among the urban and the rural populations are driven by the fact that the urban, and mostly formally employed/ working population is more likely to be educated and more exposed to the media and other modern sources of health information, including the internet.[81,82] The rural population and uneducated on the other hand, are most likely to be poor, and less likely to be exposed to print and electronic media which have been reported as major sources of information on CVDs and risk factors. The rural populations in SSA have also been shown to utilize health services less than their urban counterparts,[83,84] and rely on information from their families.[33] Exploring the determinants of health in rural areas, such as the role of the family, is therefore important if health promotion policies and strategies are to result in significant improvements in health status.

Traditionally the major sources of information on CVD, respectively CVD risk factors have been shown to include electronic and print media (television, radio, newspaper) and health workers,[85,86]. Recent studies have quoted the internet as an important source of health information, especially among urban populations, teachers and other formally employed individuals, clearly illustrating the influence of the internet in health care. This situation presents an important consideration for public health policy and resource allocation for health promotion strategies in these settings.

### Strengths and limitations

This review presents evidence regarding the knowledge and awareness of CVDs in SSA. To the best of our knowledge, this is the first systematic review of the knowledge and perceptions of CVDs in SSA. Our results are based on a systematic search of five databases, integrating both qualitative and quantitative evidence on the topic. The inclusion of qualitative studies in this review meant that research findings on perceptions towards CVDs were incorporated and contributed to our understanding of and explanation of the trends of knowledge of CVDs in this study setting. As the criteria of measurement of knowledge of CVD (risk factors) was not uniform across studies (different criteria were used for classifying knowledge into low, medium or high resulting in heterogeneity across study findings), a meta- analysis could not be conducted. As the study populations differ considerably within and between countries it is difficult to disentangle to what extent educational level or cultural or country level determine knowledge and awareness levels. Still, the qualitative synthesis of available evidence of knowledge and perceptions of and perception towards CVD risk and risk factors presented in this review should speak to the current situation as most studies were published.

## Conclusions

Generally, inadequate knowledge of CVDs and the associated risk factors continues to be one of the most important factors in determining health-seeking behaviours in SSA. Knowledge levels of CVDs, risk factors and warning signs were mainly varied by type of populations and influenced by the type of employment, education levels and place of residence. Formal workers were more aware of and knowledgeable about CVD and the risk factors compared to studies conducted within rural and urban households. What this means is that education must be tailored for different groups. One-size fits all messaging is unlikely to work. Misconceptions (damaging cultural beliefs such as witchcraft and spiritual causal theories) must be addressed in ways that enhance biomedical understandings without stigmatizing cultural understandings. Adequate attention and awareness creation on the adverse implications of CVD related risk behaviours such as smoking, alcohol consumption and sedentary lifestyle on this population cannot be overemphasized. Effective policy measures, public awareness and surveillance mechanisms that takes into consideration the socio-cultural context of these behaviours need to be developed and implemented in this region. Evidence provided in this study can guide context specific interventions, aimed at mitigating CVDs by improving levels of knowledge and awareness of the conditions and risk factors among SSA populations.

## Supporting information

S1 Text Search strategy for PubMed. (DOCX)

S1 File PRISMA 2009 checklist. (DOC)

S2 File Results of quality assessment of the quantitative and qualitative studies. (XLSX)

## Abbreviations

NCD: Non-communicable diseases
CVD: Cardiovascular disease
CHD: Coronary Heart Disease
MI: Myocardial infarction
SSA: Sub-Saharan Africa
