# Supplementary material for: Knowledge and awareness of and perception towards cardiovascular disease risk in sub-Saharan Africa: A systematic review
Source: PLoS One. 2017 Dec 12;12(12):e0189264. doi: 10.1371/journal.pone.0189264 (PMC5726714; doi:10.1371/journal.pone.0189264)
Supplement: S1 Text — (DOCX) [file pone.0189264.s001.docx]

**Search strategy**

**PubMed**

| #30,"Search (#20 OR #27) Filters: Humans Sort by: [relevance]",2192 |
| --- |
| #29,"Search (#20 OR #27) Sort by: [relevance]",3029 |
| #28,"Search #20 AND #27",158 |
| #27,"Search #21 AND #26",2815 |
| #26,"Search #22 AND #25",244406 |
| #25,"Search #23 OR #24",3554020 |
| #24,"Search Smoking OR Dyslipidemia OR Cholesterol OR Sedentary lifestyle OR Physical *activity OR Stress OR Alcohol Diabetes OR Obesity OR LDL OR Hypertension OR Blood pressure[Title/Abstract]",2183841 |
| #23,"Search Cardiovascular diseas* OR Stroke OR Ischemic heart disease OR Coronary heart disease OR Cerebrovascular OR Vascular disease OR Myocardial infarction[Title/Abstract]",1912226 |
| #22,"Search Perception* OR Awareness OR Knowledge OR Perceiv* OR Belief* OR Understanding[Title/Abstract]",1603245 |
| #21,"Search Benin OR Botswana OR Burkina Faso OR Cameroun OR Cameroon OR Congo OR Congo, Ethiopia OR Ghana OR Guinea OR Ivory Coast OR Kenya OR Lesotho OR Madagascar OR Malawi OR Mauritius OR Mozambique OR Namibia OR Niger OR Nigeria OR Rwanda OR Senegal OR Sierra Leone OR South Africa OR Swaziland OR Tanzania OR Togo OR Uganda OR Zambia[Title/Abstract]",406296 |
| #20,"Search #11 AND #19",372 |
| #19,"Search #7 AND #18",30771 |
| #18,"Search #9 OR #10",2327837 |
| #14,"Search #12 AND #13",2018 |
| #13,"Search #7 AND #10",10691 |
| #12,"Search #7 AND #9",22098 |
| #11,"Search ""Africa South of the Sahara""[Mesh]",165794 |
| #10,"Search ""Tobacco Use""[Mesh] OR ""Hyperlipidemias""[Majr]) OR ""Sedentary Lifestyle""[Majr]) OR ""Exercise""[Majr:NoExp]) OR ""Stress, Psychological""[Majr:NoExp]) AND ""Obesity, Abdominal""[Majr]) OR ""Diabetes Mellitus""[Mesh]",353269 |
| #9,"Search Cardiovascular Diseases[Mesh]",2071805 |
| #7,"Search (((""Awareness""[Mesh]) OR ""Attitude to Health""[Mesh]) OR ""Sociology, Medical""[Mesh]) OR ""Comprehension""[Majr]",358241 |
